# Supplementary figures and images for: Retrospective study on the correlation between CXCL13, immune infiltration, and tertiary lymphoid structures in cutaneous squamous cell carcinoma
Source: PeerJ. 2025 May 7;13:e19398. doi: 10.7717/peerj.19398 (PMC12065455; doi:10.7717/peerj.19398)

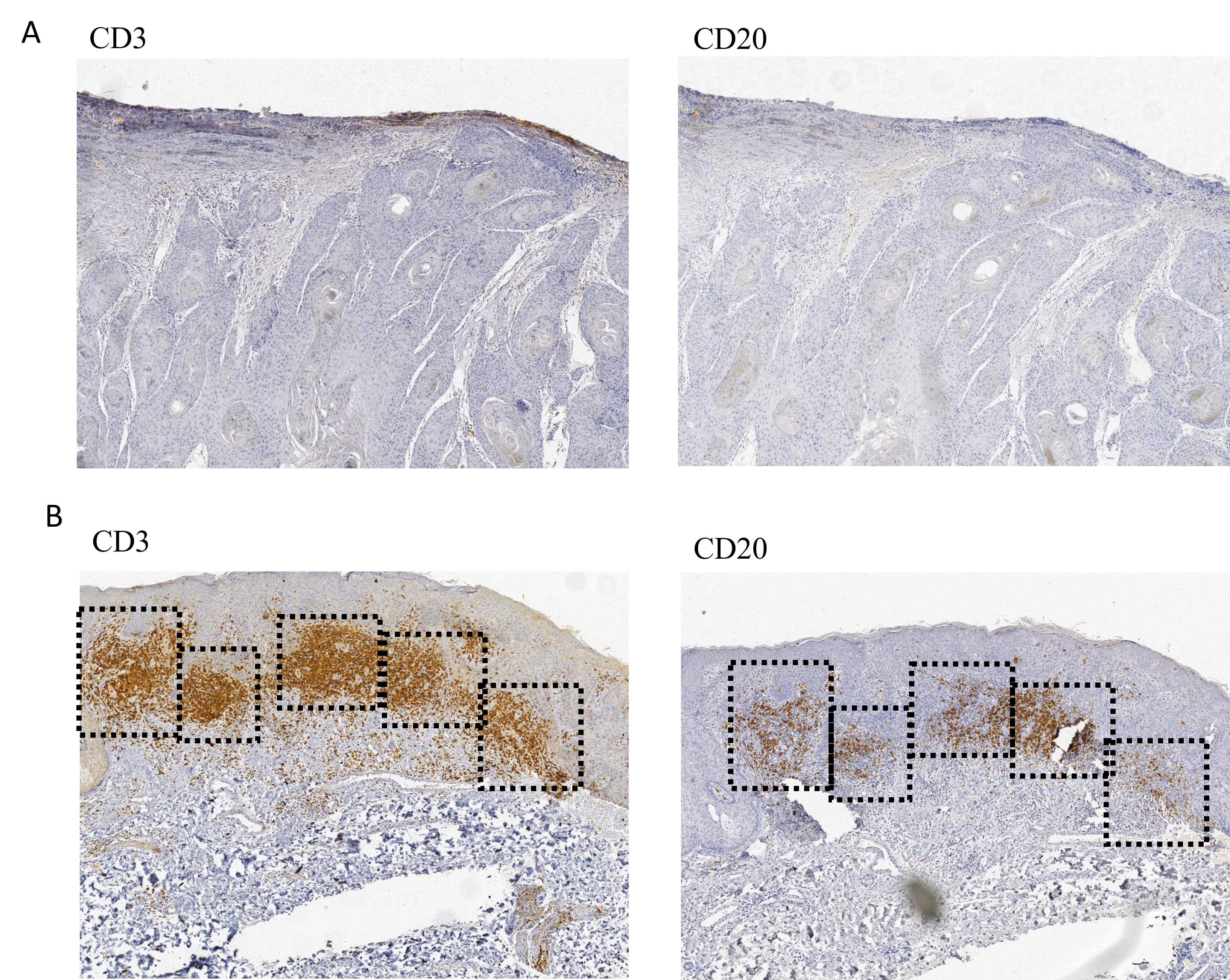

Supplement: Supplemental Information 1 — (A) Representative immunohistochemical image of tissue sections without TLS at 40x magnification. (B) Representative images showing high-density TLS (40x), outlined by squares. Five TLS are visible in this field. [file peerj-13-19398-s001.png]

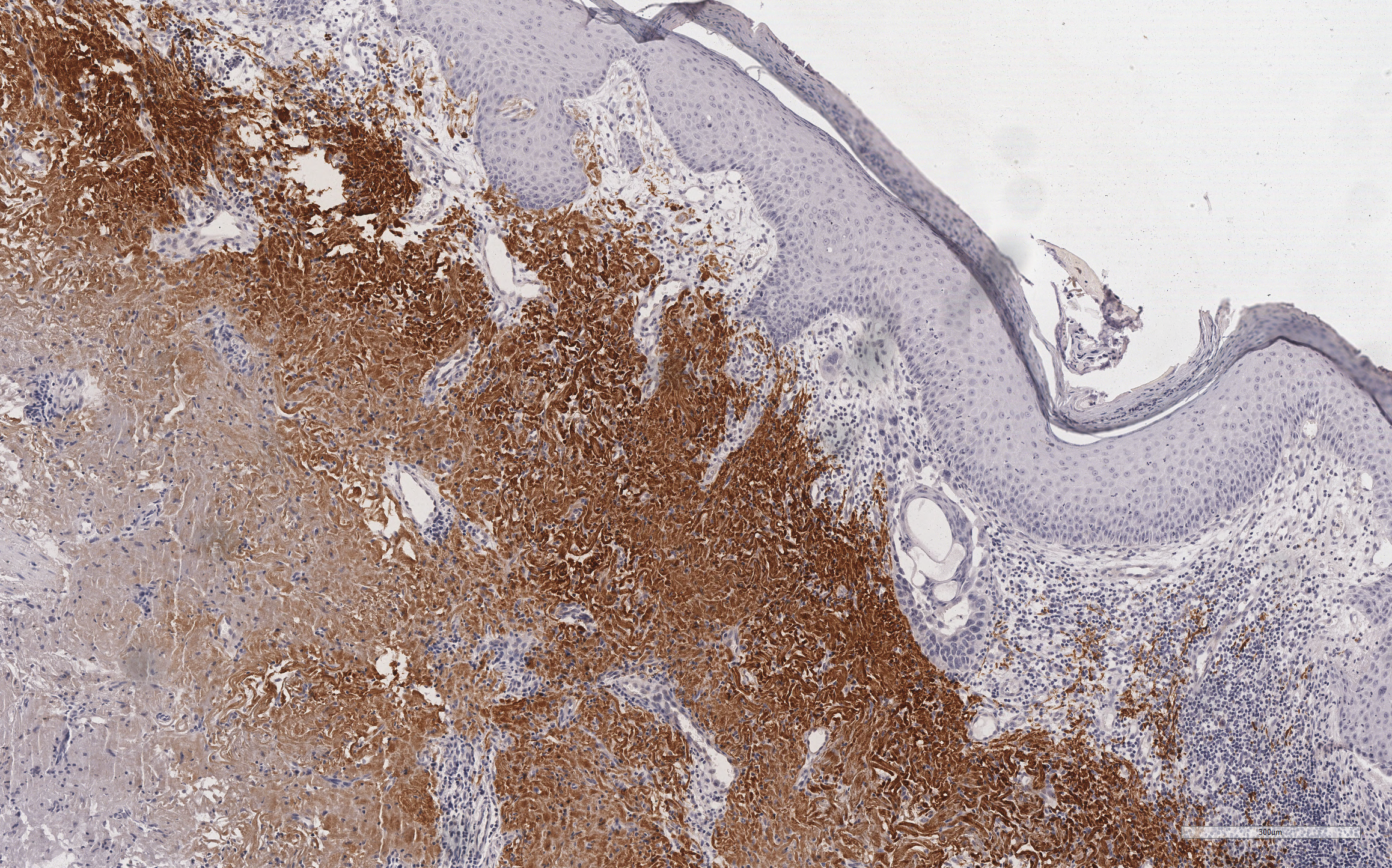

Supplement: Supplemental Information 2 — Brown staining indicates CXCL13. [file peerj-13-19398-s002.tif]

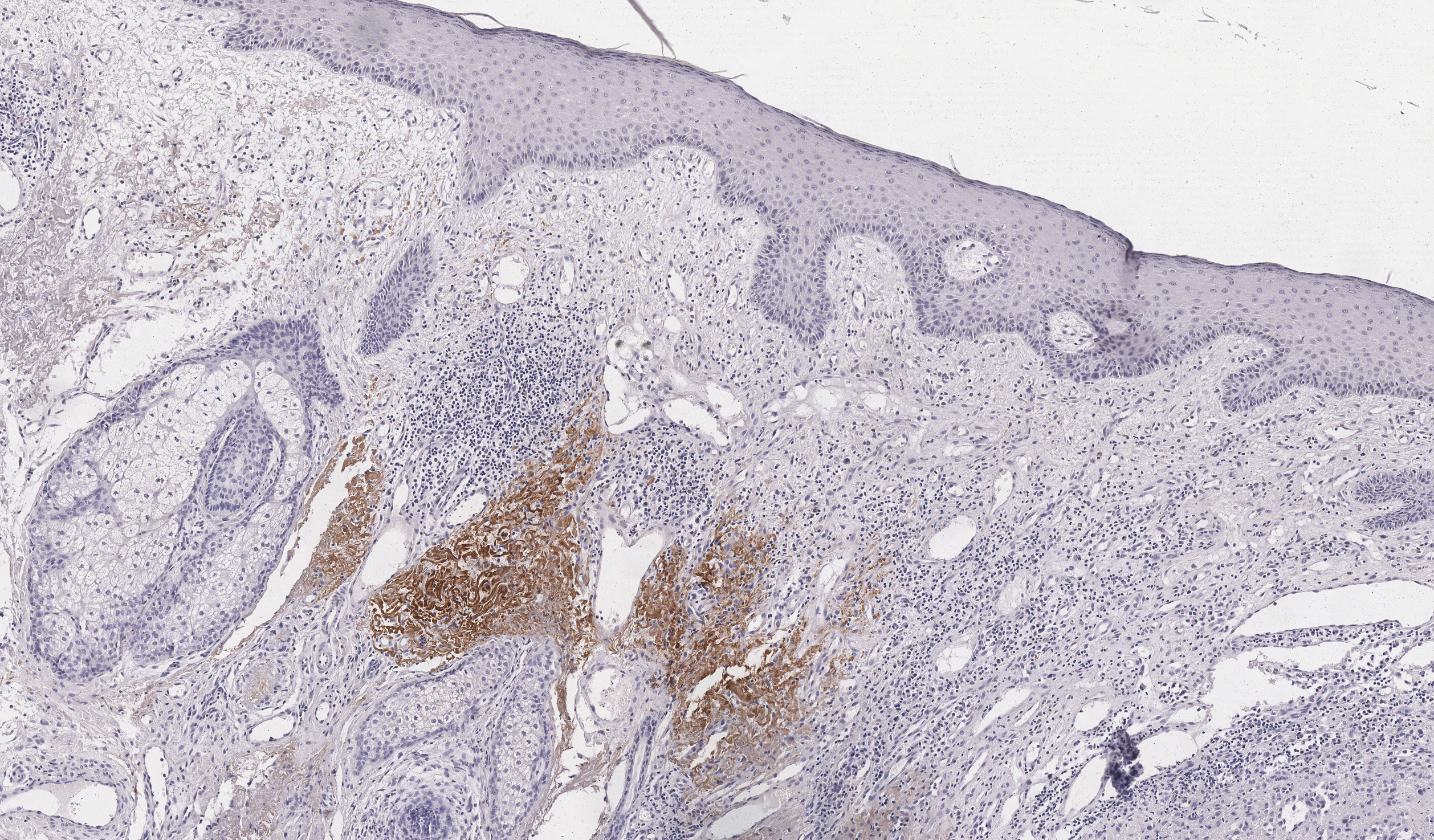

Supplement: Supplemental Information 3 — Brown staining indicates CXCL13. [file peerj-13-19398-s003.tif]
